# Weighted Single-Step Genome-Wide Association Study for Growth Traits in Chinese Simmental Beef Cattle

**DOI:** 10.3390/genes11020189

**Published:** 2020-02-11

**Authors:** Zhanwei Zhuang, Lingyang Xu, Jie Yang, Huijiang Gao, Lupei Zhang, Xue Gao, Junya Li, Bo Zhu

**Affiliations:** 1 Laboratory of Molecular Biology and Bovine Breeding, Institute of Animal Sciences, Chinese Academy of Agricultural Sciences, Beijing 100193, China; zwzhuang@outlook.com (Z.Z.); xulingyang@163.com (L.X.); gaohuijiang@caas.cn (H.G.); zhanglupei@caas.cn (L.Z.); gaoxue76@126.com (X.G.); lijunya@caas.cn (J.L.); 2 College of Animal Science and National Engineering Research Center for Breeding Swine Industry, South China Agricultural University, Guangzhou 510642, China; jieyang2012@hotmail.com

**Keywords:** Chinese Simmental beef cattle, weighted single-step GWAS, growth traits, SNP

## Abstract

Improving the genetic process of growth traits is one of the major goals in the beef cattle industry, as it can increase meat production and reduce the cost of raising animals. Although several quantitative trait loci affecting growth traits in beef cattle have been identified, the genetic architecture of these economically important traits remains elusive. This study aims to map single nucleotide polymorphisms (SNPs) and genes associated with birth weight (BW), yearling weight (YW), average daily gain from birth to yearling (BYADG), and body weight at the age of 18 months (18MW) in a Chinese Simmental beef cattle population using a weighted, single-step, genome-wide association study (wssGWAS). Phenotypic and pedigree data from 6022 animals and genotypes from 744 animals (596,297 SNPs) were used for an association analysis. The results showed that 66 genomic windows explained 1.01–20.15% of the genetic variance for the four examined traits, together with the genes near the top SNP within each window. Furthermore, the identified genomic windows (>1%) explained 50.56%, 57.71%, 61.78%, and 37.82% of the genetic variances for BW, YW, BYADG, and 18MW, respectively. Genes with potential functions in muscle development and regulation of cell growth were highlighted as candidates for growth traits in Simmental cattle (*SQOR* and *TBCB* for BW, *MYH10* for YW, *RLF* for BYADG, and *ARHGAP31* for 18MW). Moreover, we found 40 SNPs that had not previously been identified as being associated with growth traits in cattle. These findings will further advance our understanding of the genetic basis for growth traits and will be useful for the molecular breeding of BW, YW, BYADG, and 18MW in the context of genomic selection in beef cattle.

## 1. Introduction

Beef cattle provide a large proportion of the meat consumed by humans throughout the world [1]. Improving the genetic process of growth traits (e.g., body weight and average daily gain) is one of the major goals in the beef cattle breeding industry, as it can increase meat production and reduce the cost of raising animals [2,3]. The key to accelerating the progress towards this goal is to genetically select elite cattle and to mine major genes that affect growth traits. A genome-wide association study (GWAS) can detect significant single nucleotide polymorphisms (SNPs) or genomic regions that are associated with economically important traits based on the linkage disequilibrium (LD) between SNPs and possible causative mutations [4]. GWASs have recently been used to identify several quantitative trait loci (QTLs) and genes associated with growth traits in beef cattle [1,3,5]. For instance, Kim et al. [6] used 602 crossbred cattle of *Bos taurus* (Angus) and *Bos indicus* (Brahman) genotyped for 417 microsatellite markers and detected a total of 35 QTLs for growth traits (e.g., birth weight and yearling weight). Buzanskas et al. [7] performed a GWAS in 404 Canchim beef cattle using BovineHD BeadChip and found four SNPs associated with birth weight. Jahuey-Martinez et al. [8] found 18 SNPs located in 13 Bos taurus chromosomes (BTA) and highlighted five genes (*TRAF6*, *CDH11*, *KLF7*, *MIR181A-1* and *PRCP*) that were associated with growth traits in a population of 855 Charolais beef cattle genotyped for 76,883 SNPs. Although some progress has been made, the genetic architecture of these economically important traits remains poorly understood. Furthermore, the majority of GWASs for growth traits in beef cattle have only used a small sample of genotyped animals and low-density SNP arrays, which has limited the statistical power of the association analysis [1,8]. To address this issue, the weighted single-step GWAS (wssGWAS) is preferable for association analysis in Chinese beef cattle, for which large numbers of individuals have phenotypes and pedigrees but fewer are genotyped.

The wssGWAS estimates the SNP effects using genomic estimated breeding values (GEBVs) by solving a blend of pedigrees and SNPs derived matrix H, which was used in weighted single-step genomic best linear unbiased prediction (wssGBLUP). This approach can make full use of genealogical information and phenotypes of genotyped and nongenotyped animals [9]. The weighted single-step approach has been successfully applied to domesticated animals, and has led to the detection of additional QTLs and candidate genes for growth traits in Nellore cattle [3], semen traits in Duroc boar pigs [10], and milk protein composition traits in Chinese Holstein dairy cattle [11]. However, to our knowledge, few of the studies examining growth traits in Simmental beef cattle have used wssGWAS. Therefore, the objective of this study was to identify genomic regions and candidate genes associated with growth traits (birth weight (BW), yearling weight (YW), average daily gain from birth to yearling (BYADG), and body weight at the age of 18 months (18MW)) in Chinese Simmental beef cattle using the wssGWAS approach. In addition, gene enrichment analysis was performed to better understand the biological processes and pathways shared by trait-associated genes.

## 2. Materials and Methods

### 2.1. Ethics Statement

All animals used in the current study were treated following the guidelines for the care and use of experimental animals established by the Ministry of Agriculture and Rural Affairs of China. The ethics committee of the Science Research Department of the Institute of Animal Sciences, Chinese Academy of Agricultural Sciences (CAAS) (Beijing, China) approved this study. The approval ID/permit numbers are SYXK (Beijing) 2008-007 and SYXK (Beijing) 2008-008.

### 2.2. Animals, Phenotypes and Pedigree

The animals used in this study originated from 12 Chinese Simmental beef cattle core farms. These cattle were raised in different regions of China that participated in the national joint beef cattle breeding and genetic improvement program. In brief, a total of 6022 Simmental beef cattle (2878 males and 3144 females) born from 2001 to 2019 were used in this study. Among them, 6022 animals were used in wssGWAS for BW; 3996 individuals were used in wssGWAS for YW and BYADG; 3137 animals were used in wssGWAS for 18MW. Genealogical information was available for all Chinese Simmental beef cattle (both males and females). The animals born from 2018 to 2019 were only used in the BW association analysis because many of these cattle lacked phenotypic records for YW and 18MW. Yearling weight and 18MW of Simmental beef cattle was recorded at about 360 ± 30 days and 540 ± 30 days of age, respectively. Average daily gain from birth to yearling was calculated by subtracting the birth weight from the yearling weight and dividing by the number of days during this period. For the four traits under study, outliers beyond three standard deviations were removed before the association analysis.

### 2.3. Genotyping and Quality Control

A total of 744 Chinese Simmental beef cattle was genotyped using Illumina BovineHD BeadChips, which contained 777,962 SNPs. Quality control (QC) procedures were conducted using the PLINK v1.07 software (Boston, MA, USA) [12]. Individuals with call rates <95%, SNPs with minor allele frequency <0.05, call rates <95% and SNPs that failed the Hardy-Weinberg equilibrium test (*p* < 10^−6^) were removed. In addition, SNPs were also excluded if they were located on the sex chromosomes or had no positional information. After the QC, a final set of 596,297 SNPs for 744 Simmental beef cattle were retained for subsequent analyses.

### 2.4. Weighted Single-Step Genome-Wide Association Study

The wssGBLUP proposed by Wang et al. [9] was utilized to make use of all available phenotypes, pedigree, and genotypes using the BLUPF90 family programs [13]. The RENUMF90 module was used to extract data for phenotypes, pedigrees, and genomic markers in raw file format. The AIREMLF90 module was used to estimate the variance components that were used in BLUPF90 to predict GEBV. The postGSf90 module was used to conduct the wssGWAS. The four traits were analyzed using the same single trait animal model in wssGBLUP as described below:

(1) y=Wb+Za+e

where *y* represented a vector of phenotypic observations; *b* was the vector of fixed effects. In this study, sex, year of birth, use types (meat or dual-purpose), and farms were treated as fixed effects for all traits. In addition, age (in days) was included in models for YW, 18MW and BYADG as fixed effects. *a* was the vector of additive genetic effects and *e* denoted the residuals; and *W* and *Z* were the incidence matrices of *b* and *a*, respectively. It was assumed that

(2) $$a\;\sim \;N\left(0,H\sigma_{a}^{2}\right)$$

and

(3) $$e\;\sim \;N\left(0,I\sigma_{e}^{2}\right)$$

where $\sigma_{a}^{2}$ and $\sigma_{e}^{2}$ were the additive genetic variance and residual variance, respectively. *H* was a blend of pedigrees and the SNP derived matrix and *I* denoted the identity matrix. The inverse of matrix *H* was calculated as follows:

(4) $$H^{-1}=\;A^{-1}+\;\left[\begin{array}{cc} 0 & 0\\ 0 & G^{-1}-A_{22}^{-1} \end{array}\right]$$

where *A* denoted the numerator relationship matrix based on the pedigree for all individuals; *A*_22_ was the numerator relationship matrix for the genotyped animals; and the *G* matrix was a genomic relationship matrix that was constructed as described by Vanraden [14]:

(5) $$G=\frac{ZDZ^{\prime }}{\sum_{i=1}^{M}2p_{i}\left(1-p_{i}\right)}$$

where *Z* was an incidence matrix adjusted for allele frequencies, and *D* denoted a diagonal matrix of weights for SNP variances. M was the number of markers, and $p_{i}$ represented the minor allele frequency of the *i*th SNP. The SNP effects and weights for wssGWAS were calculated iteratively as follows [9]:

1. In the first iteration, set *t* = 1,
  (6) $$D_{\left(t\right)}=I$$
  (7) $$G_{\left(t\right)}=\lambda ZD_{\left(t\right)}Z^{\prime }$$
  (8) $$\lambda =\frac{1}{\sum_{i=1}^{M}2p_{i}\left(1-p_{i}\right)}$$
2. Estimate GEBV for all animals using ssGBLUP approach;
3. Compute SNP effects as
  (9) $${\hat{u}}_{\left(t\right)}=\lambda D_{\left(t\right)}Z^{\prime }G_{\left(t\right)}^{-1}{\hat{a}}_{g}$$
  where ${\hat{u}}_{\left(t\right)}$ was a vector of the SNP effects estimation and ${\hat{a}}_{g}$ was the GEBV of animals that were genotyped;
4. Calculate SNP weights for the next iteration using
  (10) $$d_{i\left(t=1\right)}={\hat{u}}_{i\left(t\right)}^{2}2p_{i}\left(1-p_{i}\right)$$
  where *i* was the *i*th SNP;
5. Normalize SNP weights to keep the total genetic variance constant as
  (11) $$D_{\left(t+1\right)}=\frac{tr\left(D_{\left(t\right)}\right)}{tr\left(D_{\left(t+1\right)}\right)}D_{\left(t+1\right)}$$
6. Calculate *G*_(*t*+1)_
  (12) $$G_{\left(t+1\right)}=\frac{ZD_{\left(t+1\right)}Z^{\prime }}{\sum_{i=1}^{M}2p_{i}\left(1-p_{i}\right)}$$
7. Set *t* = *t* + 1 and loop to step 2.

In this study, the procedure was run for three iterations as used in Wang et al. [9] and the wssGWAS results were represented by the proportion of genetic variance explained by the windows of 20 successive adjacent SNPs [15]. The percentage of additive genetic variance explained by the *i*th SNP window was calculated as:

(13) $$\frac{var\left(a_{i}\right)}{\sigma_{a}^{2}}\times 100\%=\;\frac{var\left(\sum_{j=i}^{20}z_{j}{\hat{u}}_{j}\right)}{\sigma_{a}^{2}}\times 100\%$$

where $a_{i}$ was the genetic value of the *i*th window consisting of 20 adjacent SNPs; $\sigma_{a}^{2}$ was the total genetic variance and $z_{j}$ was a vector genotype of the jth SNP for all animals; and ${\hat{u}}_{j}$ was the SNP effect of the *j*th SNP within the *i*th window. Because the windows size is 20, the proportion of variance assigned to SNP 1 is calculated from SNP 1 to 20, for SNP 2 it goes from 2 to 21, and so forth. Therefore, the SNP that contributed approximately equally to the 20-adjacent-SNP window was defined as the most important marker (top SNP).

### 2.5. Identification of Candidate Genes

Genomic windows that explained more than 1.0% of the genetic variance were selected as possible QTL regions associated with growth traits in Chinese Simmental beef cattle. Genes were searched using the Ensembl database [16] based on the SNP position that belonged to the significant genomic windows. In order to better understand the biological processes and pathways shared by these candidate genes, we conducted GO and KEGG enrichment analysis using DAVID bioinformatics resource (version 6.8) [17]. Significantly enriched terms were assessed using Fisher’s exact test (*p* < 0.05) and genes involved in biological processes were highlighted [18].

## 3. Results and Discussion

### 3.1. Descriptive Statistics and Heritabilities for the Growth Traits

Descriptive statistics of the observed phenotypes are shown in Table 1. The coefficients of variation (CV) for BW, YW, BYADG and 18MW were 11.97%, 16.36%, 18.45% and 18.61%, respectively. The results indicated that substantial phenotypic variation of these four traits exists in the Simmental beef cattle population. The heritability estimates for BW, YW, BYADG and 18MW in Chinese Simmental beef cattle were 0.42, 0.24, 0.23, and 0.43, respectively. 

### 3.2. Summary of the wssGWAS Results 

**Figure 1:**
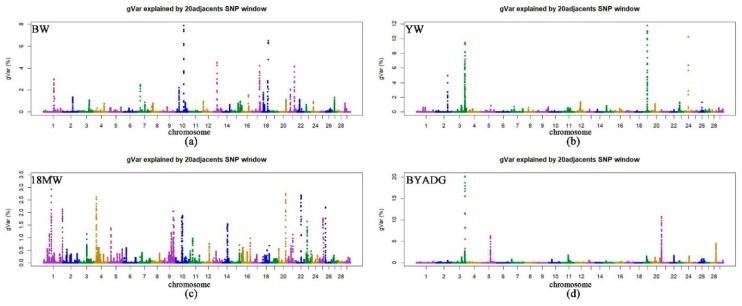
Manhattan plots for the percentage of genetic variance by 20 adjacent SNP windows for growth traits in Chinese Simmental beef cattle. gVar (%) represent the proportion of genetic variance explained by 20 adjacent SNPs. (**a**) BW: birth weight; (**b**) YW: yearling weight; (**c**) 18MW: body weight at the age of 18 months; (**d**) BYADG: average daily gain from birth to yearling.

We performed a wssGWAS in Simmental beef cattle populations to map genetic markers and genes associated with BW, YW, BYADG and 18MW. The wssGWAS results were represented by the proportion of genetic variance explained by windows of 20 successive SNPs (Figure 1). Genomic windows that explained more than 1.0% of the additive genetic variance of the four traits are shown in Table 2, Table 3, Table 4 and Table 5, together with the genes near the most important SNPs within each window. In total, 66 nonredundant windows that explained 1.01–20.15% of the additive genetic variance for the four growth traits were identified. Furthermore, the identified genomic windows explained 50.56%, 57.71%, 61.78%, and 37.82% of the genetic variances for BW, YW, BYADG, and 18MW, respectively.

### 3.3. wssGWAS for BW

Analysis was undertaken of the association with BW identified 18 genomic windows that were located on BTA1, 2, 3, 7, 10, 13, 16, 17, 18, 20, 21, 22, and 27. The identified genomic windows explained 1.07–7.89% of the additive genetic variances for BW. Genes nearest to the peak SNPs within each window were treated as potential associated candidates with BW (Table 2). Among these significant windows, the most important region was located at BTA10: 64,843,548–64,888,989 bp, which explained 7.89% of the genetic variance for BW. The gene adjacent to the top SNP, BovineHD1000018698, was sulfide quinone oxidoreductase (*SQOR*). SQOR is a protein coding gene that may interact with the inner mitochondrial membrane in a monotopic fashion and catalyze the mammalian metabolism of H_2_S (hydrogen sulfide) in human [19]. Veeranki and Tyagi et al. [20] proposed a model where H_2_S may function in skeletal muscle wasting/fibrosis as a result of metabolic complications (such as from obesity), which implied that the features of H_2_S reversed muscle damage and moderated metabolic myopathy. The second most important window (BTA18: 46,973,033–47,054,361 bp) was located inside the tubulin folding cofactor B (*TBCB*) gene, which plays a role in modulating cytoskeletal activity [21]. TBCB was proposed as a candidate gene related to meat quality in pigs due to the correlation between the protein filaments of the cytoskeleton and actin filaments [22]. In the modern beef cattle industry, meat quality traits and growth traits are two important breeding goals in genetic improvement programs. Furthermore, there is a strong genetic correlation between meat quality and growth traits and therefore, the suggestion of *TBCB* gene as a potential candidate for BW in cattle is reasonable [23]. BW is a typical polygenic quantitative trait which may be subject to nutritional intake, feeding environment of cows during pregnancy, and in some cases, sex-specific genomic imprinting [24]. Results from this study implied that genetic factors may contribute to a large share of the variation in BW in beef cattle and therefore, the identified SNPs (which explain >1% of the genetic variance) can be used for genetic improvement in the context of genomic selection (GS).

### 3.4. wssGWAS for YW and BYADG

**Figure 2:**
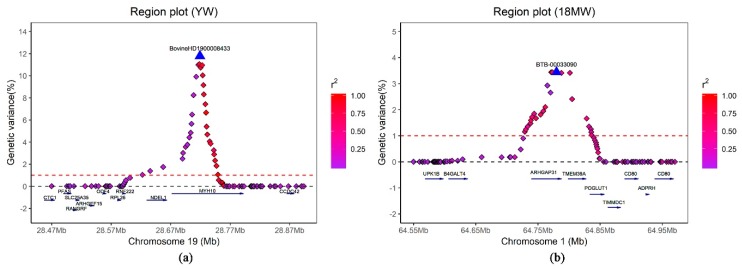
Region plots of the two major candidate regions on BTA19 and BTA1. Results were shown for YW around 28.47–28.90 Mb on BTA19 (**a**) and for 18MW at 64.55–64.98 Mb on BTA1 (**b**). The primary SNP within each region is denoted by a large blue triangle. Different levels of linkage disequilibrium between the primary SNP and surrounding SNPs were represented by colored rhombi.

In total, 14 windows in eight different chromosomes (BTA2, 3, 12, 19, 20, 23, 24 and 26) were associated with YW (Table 3). The proportion of genetic variance for these windows ranged from 1.11% to 11.80%. The most significant window (BTA19: 28,728,158–28,766,002 bp) contributed 11.80% of the genetic variance of YW. The top SNP (BovineHD1900008433) of this window was located within the myosin heavy chain 10 (*MYH10*) gene. The *MYH10* gene is a member of the myosin superfamily which shares the common features of ATP hydrolysis (ATPase enzyme activity), actin binding, and potential for kinetic energy transduction [25]. Myosin plays an important role in muscle growth and contraction [26,27]. *MYH10* is isolated from muscle cells and with functions in contractile, it is also related to myosin in nonmuscle cells [28]. Moreover, Xue et al. [29] found that *MYH10* takes part in a pathway related to growth and development in chickens and significantly upregulated the expression pattern at the transcript level. Furthermore, we evaluated the LD pattern of the SNPs in the region around 28.47–28.90 Mb on BTA19. The LD analysis revealed that the region located on *MYH10* showed a high LD level between the top SNP and nearby SNPs, implying a potential selection signature with regard to YW in Simmental beef cattle (Figure 2a). Therefore, it is reasonable to speculate that *MYH10* is a strong candidate gene for YW due to its potential roles in the genetic mechanisms of muscle development. For BYADG, 15 windows in nine different chromosomes (BTA3, 5, 11, 19, 20, 21, 22, 24 and 28) were identified (Table 4). Results showed that these windows explained 1.13–20.15% of the genetic variance for BYADG. The first three most important windows explained approximately 37.14% of the genetic variance of BYADG in total, which accounted for up to 60% of the genetic variance of all identified window interpretations. These findings implied that these windows (BTA3: 106,574,782–106,644,015 bp; BTA21: 5,941,998–5,968,820 bp; and BTA5: 77,160,030–77,212,501 bp) need more attention when selecting candidate genes for BYADG.

Notably, three windows located on BTA3 (103,471,058–103,518,431 bp, 105,080,919–105,138,584 bp, 106,574,782–106,644,015 bp) were found to be associated with both YW and BYADG, implying a pleiotropic effect for growth traits in beef cattle. Two genes which were adjacent to the top SNP within each window were mined, namely, family with sequence similarity 183 member A (*FAM183A*) and rearranged L-Myc fusion (*RLF*). *FAM183A* has been reported to play a role in autosomal recessive intellectual disability and is expressed in the human brain [30,31]. To our knowledge, few studies have clearly investigated whether *FAM183A* plays a role in influencing growth traits in domesticated animals and even in the mouse, therefore, further functional studies are required. *RLF* encodes a Zn-15 related zinc finger protein and has a general role in transcriptional regulation of fetal and adult tissues in humans [32]. *RLF* has been reported to play a role in increasing DNA methylation at a number of elements related to transcriptional regulation and is involved in maintaining epigenetic marks at CpG island shores and enhancers [33]. DNA methylation plays an essential role in embryonic muscle development and is important for the establishment and maintenance of cellular identity [34,35]. These findings could be helpful for the understanding of mechanisms of muscle development in mammalian animals.

### 3.5. wssGWAS for 18MW

Table 5 shows the 21 windows associated with 18MW which were located on BTA1, 3, 4, 5, 9, 10, 14, 20, 21, 22, 23, 25, and 26, together with 16 genes near the most important SNP within each window. The identified genomic windows explained 1.01–3.44% of the genetic variance for 18MW. The most important window, BTA1: 64788160–64867718 bp, contributed to 3.44% of the genetic variance of 18MW and was located within gene Rho GTPase activating protein 31 (*ARHGAP31*). *ARHGAP31* encodes a GTPase-activating protein (GAP) and plays a role in regulating the cellular processes of cycling between an inactive GDP-bound and active GTP-bound conformation [36]. GAP has been reported to have functions in protein trafficking and cell growth and serves as a molecular switch involved in the regulation of various cytoskeleton-related events and gene transcription [37]. Moreover, LD analysis revealed that a certain level of LD exists between the top SNP (BTB-00033090 within gene *ARHGAP31*) and its surrounding SNPs in gene transmembrane protein 39A (*TMEM39A*) (Figure 2b). The potential role of *TMEM39A* in growth needs further investigation.

### 3.6. Potential Genomic Regions and Candidate Genes Reveal the Complexity of the Genetic Architecture of Growth Traits

In an attempt to better understand the biological processes and pathways shared by the trait-associated genes, we searched 51 genes near the SNPs within each window of the four growth traits. We then performed KEGG and GO enrichment analysis. Three GO terms and no KEGG pathways were enriched for the growth traits analyzed. The enriched GO terms are involved in neuromuscular processes controlling balance (GO: 0050885) consisting of chloride intracellular channel 5 (*CLIC5*), aldehyde dehydrogenase 1 family member A3 (*ALDH1A3*), and *MYH10* genes; calcium-dependent cell-cell adhesion via plasma membrane cell adhesion molecules (GO: 0016339) consisting of cadherin 13 (*CDH13*) and neuroligin 1 (*NLGN1*) genes; motor neuron axon guidance (GO:0008045) including activated leukocyte cell adhesion molecule (*ALCAM*) and forkhead box P1 (*FOXP1*) genes. Given the potential roles of the three GO terms in biological processes, their involvement in the growth traits were further analyzed. We searched genes function based on literature reports. Then, the *MYH10*, *CDH13,* and *FOXP1* genes were highlighted as the main candidates for the growth traits of the three terms, respectively. Notably, *MYH10* gene has been highlighted as a strong candidate in the association analysis for YW and additional laboratory functional experiments would be needed. *CDH13* gene encodes a member of the cadherin superfamily and acts as a negative regulator of axon growth during neural differentiation [38]. *FOXP1* gene plays an important role in the regulation of tissue and cell type-specific gene transcription during both development and adulthood and controls adipocyte differentiation [39]. Results in GO enrichment analyses further extend to suggest that many genes are involved with growth development.

Many studies have reported QTLs and genes associated with growth traits in cattle (e.g., body weight and average daily gain) using the GWAS strategy [40,41]. However, few QTLs have consistently been identified as being associated with growth traits among breeds of cattle, including for Brangus heifers [42], Japanese Black (Wagyu) cattle [43], Charolais beef cattle [8], Siberia cattle [1], Nellore cattle [3], and the Chinese Simmental beef cattle examined in this study. These findings imply that further in-depth research is required to determine whether breed-specific QTLs exist. Despite the fact that we have recently uncovered the near-complete genome sequences of several organisms, our knowledge of the genes that underlie phenotypic differences within domestic animals remains rudimentary [44]. In particular, for complex quantitative traits, such as growth traits, the genetic basis may be subject to a number of factors including natural selection, inheritance, and evolutionary forces [45,46]. The results from our study suggest the complexity of genetic mechanisms of growth traits in Chinese Simmental beef cattle, as numerous potential genomic regions and candidate genes were associated with growth traits. Moreover, to evaluate whether SNPs associated with BW, YW, BYADG and 18MW identified in the present study correspond to any previously known QTLs, we compared the significant SNPs within each window from this study with the SNPs in the cattle QTLdb [47] based on the location of SNPs. The 40 SNPs newly found to be associated with growth traits had not been previously characterized as QTL with regard to growth in cattle (Table S1). These findings will further advance our understanding of the genetic basis for growth traits and will be useful for the molecular breeding of BW, YW, BYADG and 18MW in the context of GS in cattle.

## 4. Conclusions

In conclusion, we identified 66 nonredundant windows which explained 1.01–20.15% of the additive genetic variance for growth traits in Chinese Simmental beef cattle using the wssGWAS approach. Genes with potential functions in muscle development and regulation of cell growth were highlighted as candidates for growth traits in cattle, such as *SQOR* and *TBCB* for BW, *MYH10* for YW, *RLF* for BYADG, and *ARHGAP31* for 18MW. Specifically, the identified genomic regions will be useful for the genetic improvement of growth traits by allowing the associated SNPs to be assigned with higher weights in genomic selection.

## Figures and Tables

**Table 1:** Descriptive statistics and variance components of growth traits in Chinese Simmental beef cattle ^a^.

Traits	N	Mean	SD	Min	Max	CV (%)	$\sigma_{a}^{2}$	$\sigma_{e}^{2}$	$\sigma_{p}^{2}$	$h^{2}(\mathrm{SE})$
BW (kg)	6022	44.96	5.38	29	61	11.97	10.165	14.150	24.315	0.42 ± 0.03
YW (kg)	3996	418.21	68.37	264.94	608.65	16.36	444.650	1352.400	1797.050	0.24 ± 0.03
BYADG (kg/d)	3996	1.03	0.19	0.6	1.54	18.45	0.003	0.010	0.013	0.23 ± 0.02
18MW (kg)	3137	587.41	109.36	376.84	905.24	18.61	1193.400	1598.000	2791.400	0.43 ± 0.03

^a^ Number of animals used in the wssGWAS, $\sigma_{a}^{2}$ = genetic variance, $\sigma_{e}^{2}$ = residual variance, $\sigma_{p}^{2}$ = phenotypic variance, $h^{2}$ = heritability.

**Table 2:** Windows that explained >1% of the additive genetic variance for birth weight in Simmental beef cattle.

Chr ^a^	Window Region (bp) ^b^	gVar (%) ^c^	topSNP ^d^	Candidate Gene ^e^	Distance
10	64,843,548–64,888,989	7.89	BovineHD1000018698	*SQOR*	195,750
18	46,973,033–47,054,361	6.52	BovineHD1800013865	*TBCB*	971
13	32,898,989–32,942,587	4.51	BovineHD1300009582	*CACNB2*	106,042
17	59,422,381–59,533,974	4.23	BovineHD1700016840	*WSB2*	634
21	54,246,453–54,304,950	4.17	BovineHD2100015513	*DYNLL1*	49,145
1	86,864,010–87,000,954	3.00	BovineHD0100024730	*CCDC39*	89,435
18	43,486,335–43,539,576	2.83	BovineHD1800012856	*CEP89*	within
7	25,274,362–25,344,621	2.51	BovineHD0700006961	*CHSY3*	within
10	22,200,536–22,227,480	2.20	BovineHD4100007964	*TRDC*	within
21	18,442,926–18,507,549	2.06	BovineHD4100015053	*/*	/
18	9,390,632–9,440,030	1.78	BovineHD4100013431	*CDH13*	122,107
16	55,454,686–55,576,782	1.57	BovineHD1600015438	*BOVAGGRUS*	12,062
2	84,272,058–84,486,086	1.35	BovineHD0200024049	*SLC39A10*	461,944
27	17,515,069–17,628,482	1.35	BovineHD2700005087	*/*	/
1	93,966,859–94,071,139	1.24	BovineHD0100026650	*NLGN1*	334,630
20	58,103,188–58,151,048	1.14	BovineHD2000016087	*ANKH*	274,075
22	21,743,429–21,782,083	1.14	BovineHD2200006300	*ITPR1*	within
3	83,073,814–83,124,751	1.07	BovineHD0300023782	*ATG4C*	225,105

^a^ Chromosomes; ^b^ Window position in Ensembl; ^c^ The proportion of the genetic variance explained by 20 adjacent SNP window; ^d^ The SNP that explained the largest proportion of genetic variance within each window (equal to the value of the 20 adjacent SNP window explained); ^e^ Candidate genes near the top SNPs.

**Table 3:** Windows that explained >1% of the additive genetic variance for yearling weight in Simmental beef cattle.

Chr ^a^	Window Region (bp) ^b^	gVar (%) ^c^	topSNP ^d^	Candidate Gene ^e^	Distance
19	28,728,158–28,766,002	11.80	BovineHD1900008433	*MYH10*	within
24	30,102,158–30,164,301	10.30	BovineHD2400008151	*CHST9*	within
3	106,567,276–106,628,358	9.47	BovineHD0300030609	*RLF*	within
3	103,471,058–103,518,431	6.91	BovineHD0300029636	*FAM183A*	27,683
2	98,892,271–98,968,329	4.95	BovineHD0200028465	*CPS1*	within
3	107,082,578–107,188,510	3.09	BovineHD4100002437	*PABPC4*	1629
3	105,809,585–105,893,206	1.92	BovineHD0300030334	*CTPS*	within
3	105,103,446–105,151,204	1.57	BovineHD0300030104	*/*	/
2	98,767,231–98,852,487	1.50	BovineHD0200028425	*CPS1*	within
12	37,317,791–37,375,872	1.36	BovineHD1200010822	*ATP12A*	395,299
23	13,141,062–13,228,547	1.33	BovineHD2300003307	*KCNK17*	19,569
26	28,767,232–28,870,785	1.33	BovineHD2600007699	*SORCS1*	377,841
20	23,511,931–23,646,033	1.11	BovineHD2000007095	*SLC38A9*	within
12	37,380,083–37,454,520	1.08	BovineHD1200010844	*/*	/

^a^ Chromosomes; ^b^ Window position in Ensembl; ^c^ The proportion of the genetic variance explained by 20 adjacent SNP window; ^d^ The SNP explained that the largest proportion of genetic variance within each window (equal to the value of the 20 adjacent SNP window explained); ^e^ Candidate genes near the top SNPs.

**Table 4:** Windows that explained >1% of the additive genetic variance for average daily gain from birth to yearling in Simmental beef cattle.

Chr ^a^	Window Region (bp) ^b^	gVar (%) ^c^	topSNP ^d^	Candidate Gene ^e^	Distance
3	106,574,782–106,644,015	20.15	BTB-00148396	*RLF*	within
21	5,941,998–5,968,820	10.77	BovineHD2100001150	*ALDH1A3*	108,659
5	77,160,030–77,212,501	6.22	BovineHD0500021921	*/*	/
28	41,315,052–41,343,055	4.52	BovineHD2800011614	*WAPAL*	178,111
5	77,289,332–77,341,198	2.90	BovineHD0500021954	*/*	/
3	103,471,058–103,518,431	2.79	BovineHD0300029636	*FAM183A*	27,683
21	6,031,496–6,114,704	2.66	BovineHD2100001184	*/*	/
11	47,195,270–47,279,484	1.75	BovineHD1100013811	*RPIA*	24,890
22	30,689,705–30,734,004	1.69	BovineHD2200008831	*FOXP1*	within
3	105,080,919–105,138,584	1.58	BovineHD0300030098	*/*	/
19	24,496,287–24,525,423	1.56	BovineHD1900007093	*OR1G1*	9771
24	36,117,632–36,240,233	1.50	BovineHD2400009926	*ADCYAP1*	within
3	79,457,216–79,552,102	1.31	BovineHD0300022907	*PDE4B*	within
20	23,511,931–23,646,033	1.26	BovineHD2000007095	*SLC38A9*	within
20	63,943,581–63,965,954	1.13	BovineHD2000018191	*SEMA5A*	204,352

^a^ Chromosomes; ^b^ Window position in Ensembl; ^c^ The proportion of the genetic variance explained by 20 adjacent SNP window; ^d^ The SNP that explained the largest proportion of genetic variance within each window (equal to the value of the 20 adjacent SNP window explained); ^e^ Candidate genes near the top SNPs.

**Table 5:** Windows that explained >1% of the additive genetic variance for 18-month weight in Simmental beef cattle.

Chr ^a^	Window Region (bp) ^b^	gVar (%) ^c^	topSNP ^d^	Candidate Gene ^e^	Distance
1	64,788,160–64,867,718	3.44	BTB-00033090	*ARHGAP31*	within
20	55,978,366–56,002,160	2.74	BovineHD2000015364	*/*	/
22	32,826,296–32,856,787	2.68	BovineHD2200009375	*FAM19A4*	within
4	18,525,619–18,550,135	2.62	BovineHD0400005535	*C14H8orf59*	424,568
26	2,272,143–2,343,667	2.21	BovineHD2600000342	*/*	/
1	156,048,715–156,119,788	2.13	BovineHD0100045578	*TBC1D5*	within
9	86,926,509–86,984,196	2.06	BovineHD0900024383	*SASH1*	within
10	51,554,432–51,726,000	1.87	BovineHD1000015443	*FAM63B*	within
10	48,374,404–48,406,759	1.82	BovineHD1000014557	*VPS13C*	105,944
25	25,386,519–25,442,136	1.77	BovineHD2500007181	*KIAA0556*	29,783
9	93,609,823–93,684,438	1.72	BovineHD0900026491	*/*	/
23	19,254,288–19,290,469	1.64	BovineHD2300004888	*CLIC5*	within
14	49,154,187–49,217,544	1.54	BovineHD1400013993	*MED30*	195,164
14	47,765,008–47,835,714	1.40	BovineHD1400013511	*SAMD12*	14,727
5	18,940,413–19,018,750	1.39	BovineHD0500005477	*DUSP6*	336,392
9	71,346,676–71,377,254	1.19	BovineHD0900019755	*MOXD1*	4746
25	30,583,072–30,676,658	1.16	BovineHD2500008480	*/*	/
3	60,766,989–60,828,010	1.15	BovineHD0300018250	*TTLL7*	317,902
1	49,845,978–49,930,370	1.14	BTB-01076879	*ALCAM*	486,294
21	38,919,896–39,060,313	1.13	BTB-00818234	*/*	/
9	88,466,483–88,498,915	1.01	BovineHD0900024910	*PPP1R14C*	within

^a^ Chromosomes; ^b^ Window position in Ensembl; ^c^ The proportion of the genetic variance explained by 20 adjacent SNP window; ^d^ The SNP that explained the largest proportion of genetic variance within each window (equal to the value of the 20 adjacent SNP window explained); ^e^ Candidate genes near the top SNPs.

## Supplementary Materials

The following are available online at https://www.mdpi.com/2073-4425/11/2/189/s1, Table S1: Comparative mapping of tag SNPs with previous QTLs reported in the cattle QTL database (as of 24 November 2019) and previous GWAS results.
