# Supplementary material for: Weighted Single-Step Genome-Wide Association Study for Growth Traits in Chinese Simmental Beef Cattle
Source: Genes (Basel). 2020 Feb 11;11(2):189. doi: 10.3390/genes11020189 (PMC7074168; doi:10.3390/genes11020189)
Supplement: Supplementary file 1 [file genes-11-00189-s001.zip › Supplemental file/Supplemental Table S1.docx]

Table S1. Comparative mapping of tag SNPs with previous QTLs reported in the cattle QTL database (as of Nov 24, 2019) and previous GWAS results.

| Chr^a^ | SNP ID^b^ | Location (bp)^c^ | Trait^d^ | QTL location range (bp)^e^ | QTL ID^f^ | related QTL^g^ |
| --- | --- | --- | --- | --- | --- | --- |
| 1 | BTB-01076879 | 49845978 | 18MW | 48441931-69268709 | 7124 | Hindquarter proportions |
| 1 | BTB-00033090 | 64788160 | 18MW | 48441931-69268710 | 7125 | Hindquarter proportions |
| 1 | BovineHD0100024730 | 86864010 | BW | NA | NA | NA |
| 1 | **BovineHD0100026650** | 93966859 | BW | 70216318-94646350 | 1320 | Body weight (birth) |
| 1 | **BovineHD0100045578** | 156048715 | 18MW | 156005880-156999077 | 35215 | Average daily gain |
| 2 | **BovineHD0200024049** | 84272058 | BW | 49295365-85095065 | 10670 | Body weight (birth) |
| 2 | BovineHD0200028425 | 98767231 | YW | NA | NA | NA |
| 2 | BovineHD0200028465 | 98892271 | YW | NA | NA | NA |
| 3 | **BovineHD0300018250** | 60766989 | 18MW | 60193295-61066402 | 106628 | Body weight (weaning) |
| 3 | BovineHD0300022907 | 79457216 | BYADG | 79296224-79497422 | 4342 | Dry matter intake |
| 3 | **BovineHD0300023782** | 83073814 | BW | 82446168-83246358 | 10702 | Body weight (yearling) |
| 3 | BovineHD0300029636 | 103471058 | BYADG | NA | NA | NA |
| 3 | BovineHD0300030098 | 105080919 | BYADG | NA | NA | NA |
| 3 | BovineHD0300030104 | 105103446 | YW | NA | NA | NA |
| 3 | BovineHD0300030334 | 105809585 | YW | NA | NA | NA |
| 3 | BovineHD0300030609 | 106567276 | YW | NA | NA | NA |
| 3 | BTB-00148396 | 106574782 | BYADG | NA | NA | NA |
| 3 | BovineHD4100002437 | 107082578 | YW | 106930563-107404279 | 106522 | Milk yield |
| 4 | BovineHD0400005535 | 18525619 | 18MW | NA | NA | NA |
| 5 | **BovineHD0500005477** | 18940413 | 18MW | 18794396-60726353 | 20553 | Body weight (slaughter) |
| 5 | BovineHD0500021921 | 77160030 | BYADG | 76533399-93514025 | 56572 | Milk myristic acid content |
| 5 | BovineHD0500021954 | 77289332 | BYADG | 76533399-93514026 | 56573 | Milk myristic acid content |
| 7 | **BovineHD0700006961** | 25274362 | BW | 23405570-27437470 | 10793 | Body weight (birth) |
| 9 | BovineHD0900019755 | 71346676 | 18MW | 30894099-74904058 | 10293 | Non-return rate |
| 9 | **BovineHD0900024383** | 86926509 | 18MW | 86573306-88076670 | 106641 | Body weight (weaning) |
| 9 | BovineHD0900024910 | 88466483 | 18MW | 88436966-88849796 | 106420 | Length of productive life |
| 9 | BovineHD0900026491 | 93609823 | 18MW | 88955721-98245564 | 15182 | Calving index |
| 10 | BovineHD4100007964 | 22200536 | BW | NA | NA | NA |
| 10 | BovineHD1000014557 | 48374404 | 18MW | 38433107-52183478 | 10875 | Height (yearling) |
| 10 | BovineHD1000015443 | 51554432 | 18MW | 38433107-52183479 | 10876 | Height (yearling) |
| 10 | BovineHD1000018698 | 64843548 | BW | 58095959-67861579 | 4872 | Shear force |
| 11 | **BovineHD1100013811** | 47195270 | BYADG | 45720847-57712168 | 10902 | Body weight (yearling) |
| 12 | **BovineHD1200010822** | 37317791 | YW | 36550507-43232835 | 15733 | Body weight (slaughter) |
| 12 | **BovineHD1200010844** | 37380083 | YW | 36550507-43232836 | 15734 | Body weight (slaughter) |
| 13 | **BovineHD1300009582** | 32898989 | BW | 22480878-33021666 | 10944 | Body weight (weaning) |
| 14 | BovineHD1400013511 | 47765008 | 18MW | 47761523-47910502 | 3714 | Milk protein percentage |
| 14 | BovineHD1400013993 | 49154187 | 18MW | 48468784-50201449 | 31028 | Intramuscular fat |
| 16 | BovineHD1600015438 | 55454686 | BW | NA | NA | NA |
| 17 | BovineHD1700016840 | 59422381 | BW | NA | NA | NA |
| 18 | BovineHD4100013431 | 9390632 | BW | 7176419-25971941 | 1336 | Carcass weight |
| 18 | BovineHD1800012856 | 43486335 | BW | 11438802-46178647 | 18469 | Somatic cell score |
| 18 | BovineHD1800013865 | 46973033 | BW | NA | NA | NA |
| 19 | BovineHD1900007093 | 24496287 | BYADG | NA | NA | NA |
| 19 | BovineHD1900008433 | 28728158 | YW | 28671067-50583438 | 22873 | Intramuscular fat |
| 20 | **BovineHD2000007095** | 23511931 | BYADG | 22679451-32852577 | 11104 | Body weight (mature) |
| 20 | BovineHD2000015364 | 55978366 | 18MW | 55225858-60391422 | 172175 | Milk C14 index |
| 20 | BovineHD2000016087 | 58103188 | BW | 58002823-58996938 | 35237 | Efficiency of gain |
| 20 | BovineHD2000018191 | 63943581 | BYADG | NA | NA | NA |
| 21 | **BovineHD2100001150** | 5941998 | BYADG | 3827974-16536969 | 22762 | Body weight (birth) |
| 21 | **BovineHD2100001184** | 6031496 | BYADG | 3827974-16536970 | 22763 | Body weight (birth) |
| 21 | **BovineHD4100015053** | 18442926 | BW | 14974465-19106297 | 11121 | Body weight (yearling) |
| 21 | **BTB-00818234** | 38919896 | 18MW | 34593345-43259619 | 11127 | Body weight (weaning) |
| 21 | BovineHD2100015513 | 54246453 | BW | 53373521-58604263 | 172177 | Milk capric acid content |
| 22 | **BovineHD2200006300** | 21743429 | BW | 9425005-22132731 | 11139 | Body weight (yearling) |
| 22 | **BovineHD2200008831** | 30689705 | BYADG | 25594441-34111868 | 11149 | Body weight (yearling) |
| 22 | **BovineHD2200009375** | 32826296 | 18MW | 25594441-34111868 | 11149 | Body weight (yearling) |
| 23 | **BovineHD2300003307** | 13141062 | YW | 598091-17515851 | 1412 | Body weight (slaughter) |
| 23 | BovineHD2300004888 | 19254288 | 18MW | 19248715-19386427 | 16053 | Interval to first estrus after calving |
| 24 | **BovineHD2400008151** | 30102158 | YW | 30004563-41590595 | 11197 | Body weight (yearling) |
| 24 | BovineHD2400009926 | 36117632 | BYADG | NA | NA | NA |
| 25 | **BovineHD2500007181** | 25386519 | 18MW | 21632493-27460729 | 11209 | Body weight (weaning) |
| 25 | BovineHD2500008480 | 30583072 | 18MW | NA | NA | NA |
| 26 | **BovineHD2600000342** | 2272143 | 18MW | 14558-2378905 | 11221 | Body weight (yearling) |
| 26 | **BovineHD2600007699** | 28767232 | YW | 25910108-30988113 | 11237 | Body weight (weaning) |
| 27 | **BovineHD2700005087** | 17515069 | BW | 11798160-18729106 | 11249 | Body weight (birth) |
| 28 | BovineHD2800011614 | 41315052 | BYADG | NA | NA | NA |

^a^ Bovine autosomes ^b^ The bold data indicate previously discovered SNPs associated with growth traits in cattle ^c^ SNP location in Ensembl ^d^ Growth traits that the SNP associated with in this study ^e^ Location range of the mapped QTL in the QTL database ^f^ Identity of QTL in the cattle QTL database or published literature ^g^ The name of mapped QTL in the database (https://www.animalgenome.org/cgi-bin/QTLdb/BT/index).
